# A rare case of desmoid fibromatosis of the transverse colon mimicking a perforated malignancy

**DOI:** 10.1093/omcr/omab031

**Published:** 2021-06-18

**Authors:** Isran Ali Shah, Sheza Arif Toor, Ioannis Gerogiannis

**Affiliations:** Department of General and Emergency Surgery, Kingston Hospital NHS Foundation Trust, Kingston, Greater London, UK

**Keywords:** abdominal fibromatosis, aggressive fibromatosis, desmoid, intestinal perforation, colonic neoplasms

## Abstract

Desmoid tumour of the colon is a very rare and aggressive type of intra-abdominal desmoid fibromatosis. Patients can present with a range of symptoms from a mild chronic abdominal pain to those of an acute abdomen. We present a rare case of abdominal fibromatosis that presented as a rapidly growing mass with free intraperitoneal gas. Intraoperatively however, we found a large tumour arising from the wall of the transverse colon and local necrosis. No bowel perforation was noted. The tumour was removed with a wide resection of transverse colon’s wall instead of colectomy. The histopathology reported benign fibromatosis and excluded malignancy.

## INTRODUCTION

Desmoid fibromatosis (DF) is a rare condition that can involve many organs of the human body including intrabdominal ones. Histologically, fibromatosis is an abnormal proliferation of myofibroblasts [1]. Intra-abdominal desmoid tumours (DTs) are very rare and usually occur in patients with familial adenomatous polyposis [2]. This is a report of a rare case of benign DT of the colon presenting as a perforated malignancy and being treated surgically with complete resection.

## CASE REPORT

**Figure 1:**
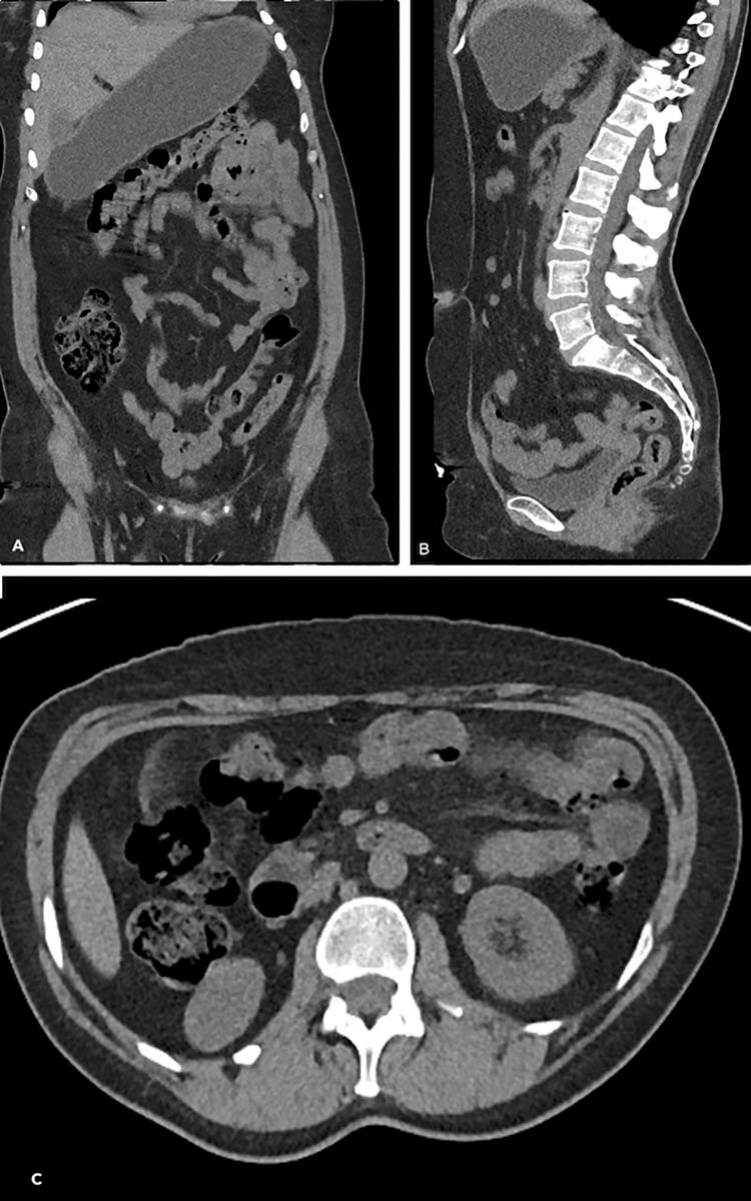
The CT scan abdomen that patient had 6 months prior to the presentation to the ED with renal colic. (**A**) Coronal, (**B**) sagittal and (**C**) axial. There is no evidence of tumour in the transverse colon.

A 51-year-old lady presented to the emergency department (ED) with generalized abdominal pain. The patient did not report any associated symptoms. She had had similar episodes in the recent past, but the symptoms were minor and resolved on their own. Her past medical history included a recent admission to ED with renal colic and a computed tomography scan (CT) of the abdomen which was clear (Fig. 1). She did not report any weight loss. The abdominal examination revealed tenderness, mainly in the left side of the abdomen, but no signs of peritonitis.

**Figure 2:**
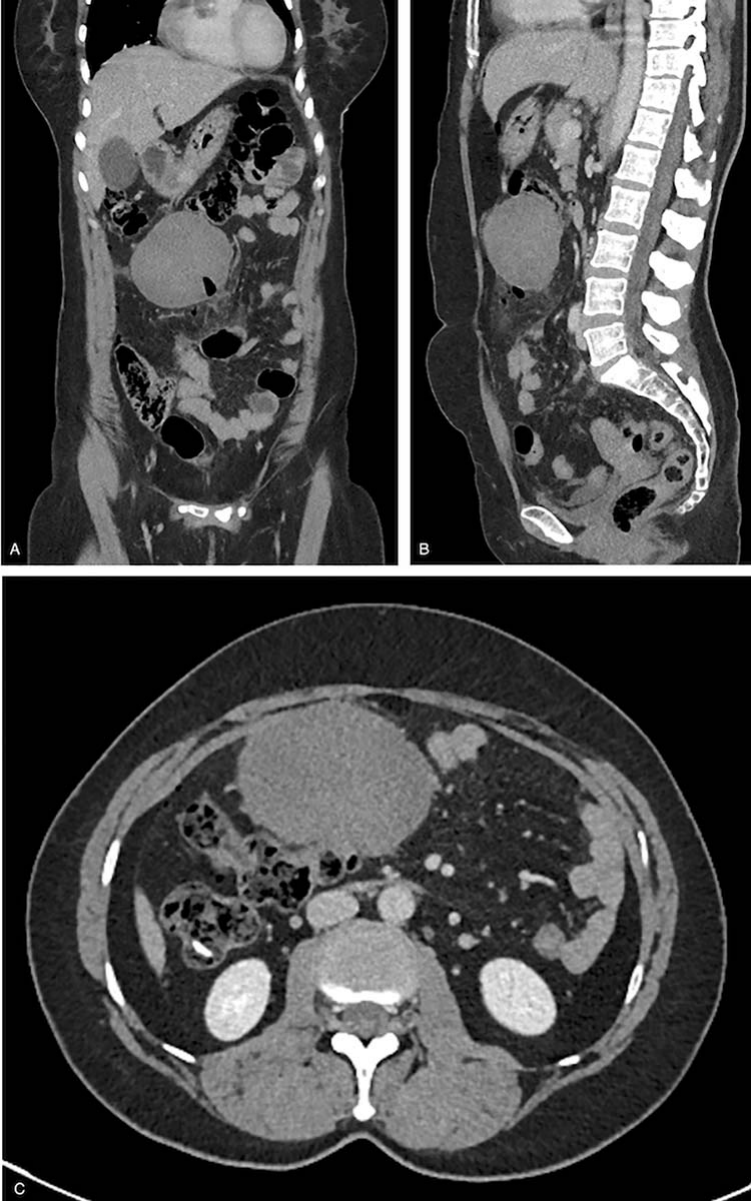
CT of the abdomen and pelvis preoperatively. (**A**) Coronal, (**B**) sagittal and (**C**) axial. The location and the size of the tumour are obvious. Also, the locules of gas are seen in (A) and (B).

Blood tests showed a C-reactive protein of 24 mg/l and white blood cells of 12 000/ml. The rest of the values were unremarkable, including lactates. She was tachycardic, but she was haemodynamically stable. Chest X-ray showed no free air under diaphragm. A CT abdomen/pelvis showed free intraperitoneal gas indicating a hollow viscus perforation. A 9.2 × 6.1 cm lesion in the mesentery, which at that time was of uncertain aetiology, was also found. The report mentioned that although the mass was inseparable from the transverse colon superiorly, it did not appear to arise from this and maintained a clear plane to the small bowel loops. The mass was of heterogeneous attenuation measuring up to 50 Hounsfield units. Further review of the scans with the consultant radiologist suggested the presence of a possible gastrointestinal stromal tumour or a desmoid (Fig. 2).

**Figure 3:**
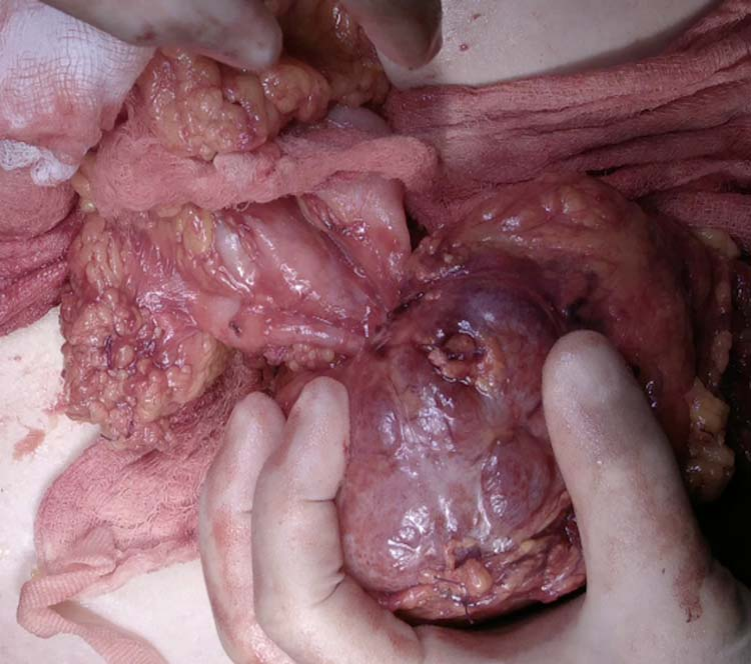
The tumour was arising from the antimesenteric border of the transverse colon.

**Figure 4:**
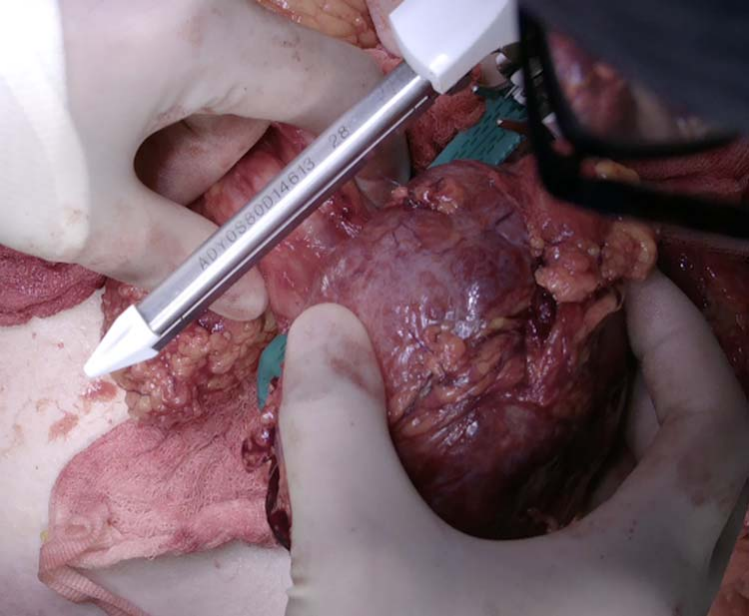
Resection of the tumour with a linear staple (80 mm) that was applied on the transverse colon.

**Figure 5:**
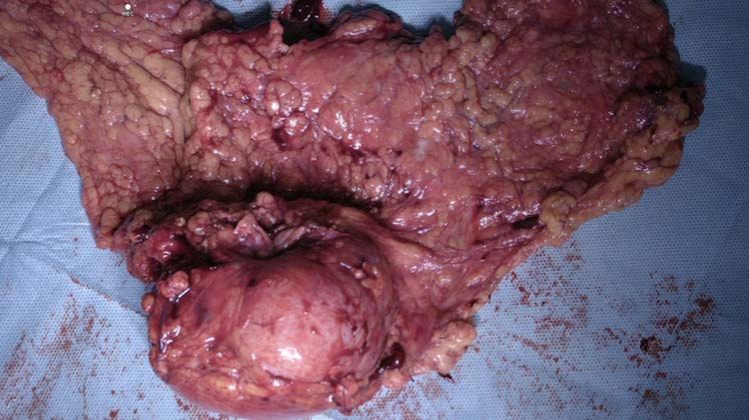
Specimen. Posterior side. En bloc resection of the tumour with the part of the colonic wall and the omentum that was coveringit.

Subsequent to the imaging and clinical findings, a decision for surgical exploration was made. A midline laparotomy was performed and a large tumour was discovered. Its wall was well defined, and it was arising from the wall of the transverse colon. The omentum was covering a part of the tumour that looked necrotic (Fig. 3). As the tumour seemed extraluminal and benign, it was decided to proceed with wide resection of the wall of the colon. A linear staple (80 mm) was applied on the transverse colon (Fig. 4), and the tumour was removed en bloc with the part of the colonic wall and the omentum that was covering it (Fig. 5). The rest of the intraperitoneal cavity was explored for possible deposits or tumours, and it was negative.

The histopathology confirmed benign fibromatosis. Macroscopically, the specimen was described as a 90 × 85 × 60 mm round mass of white tissue with attached omental fat 310 × 170 × 25 mm with no evidence of perforation. Microscopically, a spindle cell lesion composed of fascicles in places with a perpendicular pattern was revealed. The tumour cells showed diffuse nuclear staining for beta catenin and they were negative for S100, CD34, CD117 and desmin. Mitoses were seen scattered and typical in morphology excluding colonic malignancy. It was a margin-negative (R0) resection.

The patient had an uneventful post-operative period. The case was discussed in our multidisciplinary team meeting (MDT), and it was decided to continue follow-up with interval scans without chemoradiotherapy. The CT scans 6 and 12 months post-operatively showed no recurrence or signs of metastatic disease or lymphadenopathy.

## DISCUSSION

The word desmoid comes from the Greek word desmos, which means tendon-like [3]. These are locally aggressive tumours that histopathologically contain of proliferating myofibroblasts which make them solid and tough. They can be classed as extra-abdominal, intra-abdominal and within the abdominal wall. They are more common in the female gender, and evidence suggests that oestrogen levels effect their growth. Although DTs do not metastasize, their recurrence rate is high [4].

We present the first case report in literature describing a DT arising from the transverse colon of a patient who presented with acute abdomen and underwent an emergency laparotomy and wide resection without extended colectomy. There are three case reports in literature describing a colonic DT, one was a finding in an elective and two in an emergency setting, but in all of those cases, patients finally underwent a hemicolectomy [1, 5, 6].

DF remains an enigmatic disease with a variable course that can range from an incidental small tumour that can remain small and stable or become large and grow rapidly, causing death in a matter of months or years [3]. In our case, there was no evidence seen in the CT scan that was done 6 months prior to the patient’s presentation in the ED. The latter, together with the large size of the tumour (9.2 × 6.1 cm), means that its growth was rapid. Furthermore, DT can become mobile as they are attached or originated from the mesentery. Therefore, a characteristic of the DT of the abdominal cavity is that they can potentially remain asymptomatic for a long period of time before becoming symptomatic [5].

In our case, a colonic DT mimicking a hollow viscus perforation can be anticipated as diagnosis. Even though free intraperitoneal gas had been seen in the CT, no bowel perforation was noted intraoperatively. As the tumour had a necrotic area, covered with omentum, it seems that the gas was originating from the tumour necrosis. Finally, no perforation was described macroscopically in the histopathology report.

The surgical management of gastrointestinal fibromatosis is governed by the location, size and local invasion of the tumour. R0 resection has historically been the gold standard for the treatment of abdominal wall and intra-abdominal DTs; however, microscopically positive margin resection is acceptable, as recurrence rates may not be significantly affected [2]. In our case, it was a R0 resection with >1 cm margin, and after 1 year, there was no sign of recurrence. It is important that we did not have to proceed with extensive colonic resection and anastomosis. However, the intraoperative decision to proceed with a colectomy or local excision is a challenge. A preoperative and or an intraoperative discussion with a colorectal specialist is ideal even though this is not always feasible in an emergency situation. In case of a doubt or limited experience in colorectal surgery, the operating surgeon should proceed with colectomy in order to ensure negative resection margins.

DT of the colon is a very rare and aggressive type of intra-abdominal DF. Patients with colonic DT can present with a range of symptoms from a mild chronic abdominal pain to those of an acute abdomen. CT is an essential tool for the final diagnosis. The surgeon should review the scans together with the consultant radiologist in order to form the best plan preoperatively and intraoperatively. Complete resection of the tumour with negative margins macroscopically prevents local recurrence. The decision of an extended resection of the involved bowel or just local excision should be made intraoperatively depending on the tumour’s location, local invasion and surgeon’s experience. An MDT discussion, as well as follow-up, is mandatory.

## CONFLICT OF INTEREST STATEMENT

None declared.

## FUNDING

None to declare.

## ETHICAL APPROVAL

All procedures performed in studies involving human participants were done in accordance with the ethical standards of the institutional and/or national research committee and with the 1964 Helsinki Declaration and its later amendments or comparable ethical standards.

## INFORMED CONSENT

Informed consent was obtained from all individual participants included in the study. Full verbal and written informed consent has been obtained from the patient for submission of this manuscript for publication.

## GUARANTOR

Mr Ioannis Gerogiannis is acting as a guarantor for this manuscript.
